# Nocturnal salivary cortisol and perceived stress in schoolteachers: a pilot study

**DOI:** 10.3389/fpsyt.2026.1772876

**Published:** 2026-03-10

**Authors:** Pablo A. Lizana, Leandro Gálvez-Ojeda, Alexies Dagnino-Subiabre, Lydia Lera, Ivanka Kuehnel-Carreño, Gerard Clarke, Javier A. Bravo, Marcela Julio-Pieper

**Affiliations:** 1 Laboratory of Epidemiology and Morphological Sciences, Instituto de Biología, Pontificia Universidad Católica de Valparaíso, Valparaíso, Chile; 2 Center for Interdisciplinary Research in Biomedicine, Biotechnology and Well-Being (CID3B), Pontificia Universidad Católica de Valparaíso, Valparaíso, Chile; 3 Laboratory of Stress Neurobiology, Interdisciplinary Centre for Health Studies (CIESAL), Institute of Physiology, Faculty of Sciences, Universidad de Valparaíso, Valparaíso, Chile; 4 Millennium Institute for Depression and Personality Research (MIDAP), Santiago, Chile; 5 Latin Division, Keiser University, Online Education, Fort Lauderdale, FL, United States; 6 Grupo de NeuroGastroBioquıímica, Instituto de Química, Pontificia Universidad Católica de Valparaíso, Valparaíso, Chile; 7 Department of Psychiatry and Neurobehavioral Science & APC Microbiome Ireland, University College Cork, Cork, Ireland

**Keywords:** DASS-21, mental health, perceived stress, salivary cortisol, teachers

## Abstract

Teaching is characterized by high exposure to occupational stressors that negatively impact the mental health of education professionals. In this context, the present cross-sectional study aimed to analyze the relationship between salivary cortisol levels and perceived stress in a sample of teachers. Subjective stress perception was assessed using the DASS-21 questionnaire, while salivary cortisol samples were collected by the participants themselves before bedtime (PM cortisol). To examine the relationship between cortisol concentrations and the DASS-21 dimensions (depression, anxiety, and stress), non-parametric correlation analyses were applied. Results showed that 18.5% of teachers exhibited symptoms of depression, 59.3% anxiety, and 40.7% stress. Moreover, a significant positive correlation was observed between PM salivary cortisol levels and the perceived stress dimension (Rho = 0.45, p < 0.05). No significant correlations were found between cortisol and the depression or anxiety dimensions. In conclusion, the findings provide evidence of a significant association between perceived stress and the physiological response measured through PM salivary cortisol, supporting the use of this biomarker as an objective indicator of stress in educational settings. These results highlight the need to implement prevention, monitoring, and intervention strategies that integrate both psychological and physiological aspects of teacher stress.

## Introduction

Teaching has been reported as one of the most stressful professions worldwide (1, 2). Various studies have demonstrated that occupational stress is a common phenomenon in the educational field, affecting both the physical and mental health of teachers (3, 4) and often leading to burnout (5). This negative impact can manifest in the development of psychological disorders and the deterioration of professional performance (6). In this context, teacher health and well-being emerge as key factors that directly influence the quality of the teaching-learning process and job satisfaction (7, 8). Conceptually, occupational stress can be framed as a process that differentiates between exposure to work-related stressors (objective demands and contextual conditions) and the individual’s appraisal of those demands (perceived stress severity and controllability). This distinction is central because individuals facing similar objective demands may report markedly different perceived stress, with downstream consequences for health and functioning (9, 10).

From a biopsychosocial perspective, stress-related appraisals are expected to shape neuroendocrine activation, including hypothalamic–pituitary–adrenal (HPA) axis responding, thereby providing a rationale for integrating self-report measures with physiological indicators such as cortisol (11, 12). Chronic stress exposure can produce cumulative multisystem physiological strain, commonly conceptualized as allostatic load, and may progress to allostatic overload when adaptive systems are persistently challenged beyond regulatory capacity (13). Mechanistically, repeated activation of stress-response systems coupled with insufficient recovery can accumulate across time, increasing allostatic load; this is particularly relevant for teachers given the daily, recurring nature of classroom and administrative demands and the frequent extension of work into the evening (14–17). In educators, this type of stress represents a constant challenge that affects both professional performance and classroom climate (18). Teachers often experience high levels of stress due to multiple factors, including pressure to meet academic standards, classroom behavior management, administrative workload, lack of resources, and limited institutional support (19). Those reporting higher levels of occupational stress tend to exhibit lower job satisfaction and commitment, higher intention to leave the profession, and reduced capacity to provide emotional and academic support to their students (20). Accordingly, persistent occupational demands may contribute to allostatic load over time, a mechanism of “wear and tear” that is relevant for teachers given the sustained, repetitive nature of work stressors across the academic year (13, 21). Therefore, understanding the interaction between perceived stress and physiological responses is essential for designing effective monitoring and intervention strategies that promote teacher well-being. In this framework, salivary cortisol emerges as an objective and non-invasive biomarker that allows the assessment of hypothalamic–pituitary–adrenal (HPA) axis activity, the body’s main stress response system (22–24). Measuring cortisol levels in saliva is a valuable tool to understand how perceived stress relates to physiological stress-related activity, facilitating the identification of teachers at risk of developing health alterations associated with chronic stress (25). Moreover, nocturnal salivary cortisol has been proposed as a useful non-invasive marker for assessing hypercholesterolemia in different populations (26, 27).

Although associations between perceived stress and cortisol have been examined across multiple populations, less attention has been paid to nocturnal salivary cortisol (pre-bedtime), despite its relevance as an index of end-of-day HPA-axis downregulation and recovery (28). Importantly, perceived stress may be more proximally linked to end-of-day physiological activation than objective exposure alone, because it captures the subjective intensity and ongoing cognitive-emotional engagement with occupational demands, which can interfere with recovery processes (11, 12). Cortisol levels assessed near an individual’s habitual bedtime may capture sustained physiological activation during the transition to sleep, a period that is particularly sensitive to prolonged work-related demands and has been linked to sleep-related hyperarousal (29, 30). In schoolteachers, work often extends into the evening (e.g., grading and lesson preparation), making bedtime cortisol a theoretically meaningful and practically feasible biomarker for field-based occupational surveillance (31). Given these conceptual and methodological considerations, a pilot field design is appropriate to test the feasibility of bedtime sampling under real school conditions and to generate preliminary estimates of association that can guide adequately powered future studies. Therefore, the aim of the present study was to analyze the relationship between nocturnal salivary cortisol levels and perceived stress in teachers through a cross-sectional study conducted in school settings to evaluate the utility and feasibility of bedtime salivary cortisol as an end-of-day biomarker of stress-related HPA activity in schoolteachers.

## Methodology

The study employed a pilot field and cross-sectional design in school settings to examine the association between perceived stress and pre-bedtime salivary cortisol under real-world conditions. Participant selection was conducted using a non-probabilistic sampling method, aiming to ensure sample representativeness in terms of gender. Two primary instruments were used for data collection: a perceived stress questionnaire and salivary cortisol measurement. The Depression, Anxiety, and Stress Scale (DASS-21) was applied to assess teachers’ subjective perception in the dimensions of depression, anxiety, and stress. This 21-item self-administered instrument has been validated in Chile (32) and previously used in Chilean schoolteacher populations (4). The DASS-21 comprises three subscales evaluating negative emotional states of depression, anxiety, and stress, each with seven items and four-point Likert response options (0 = “Did not apply to me at all” to 3 = “Applied to me very much or most of the time”). Symptom level classification used cut-off points proposed by Lovibond & Lovibond (33), defining the ranges as: no symptoms, mild, moderate, severe, and extremely severe (4, 34). Internal consistency of the DASS-21 dimensions was evaluated using Cronbach’s alpha coefficient, with values ≥ 0.70 considered acceptable (35).

Saliva samples were collected by participants themselves before bedtime using Salivette devices (Sarstedt, Germany). Participants were allowed to follow their normal bedtime patterns, however they were instructed to avoid eating or drinking during the 15 minutes prior to sample collection and not to brush their teeth at least one hour before. Once obtained, samples were immediately stored at 4 °C until collected by the research team. Saliva was then extracted by centrifugation at 1000 g for 5 minutes and stored at –20 °C. Salivary cortisol concentrations were determined by ELISA (ALPCO, USA), following the manufacturer’s instructions. The assay detection limit was 0.128 nmol/mL, with intra-assay and inter-assay coefficients of variation of 4.7% and 6.9%, respectively. Samples were diluted 1:2 in assay buffer and analyzed in duplicate; only results with variability below 10% between duplicates were considered. Analyses were repeated up to a third time when variability was higher than 10%. Results were expressed in nmol/L, using <6 nmol/L as the cut-off point for nocturnal salivary cortisol (36).

Statistical analysis was conducted using STATA 16 for Windows. Data normality was assessed using the Shapiro–Wilk test. To analyze the relationship between salivary cortisol levels and the DASS-21 dimensions (depression, anxiety, and stress), Spearman correlation was used due to non-normal distribution of the variables. In addition, a sensitivity analysis was performed for extreme data. For the stress dimension, the correlation analysis was performed without the extreme data point (DASS-21 stress score = 20).

## Results

A total of 31 teachers were recruited, of whom 4 were excluded due to differences greater than 10% between duplicates in salivary cortisol measurement. Therefore, 27 participants were included in the analysis, of which 85.2% (n = 23) were women, with a mean age of 39.6 ± 14.4 years. The internal consistency of the DASS-21 was adequate, with Cronbach’s alpha values of 0.79 for depression, 0.78 for anxiety, and 0.85 for stress. The average scores of the DASS-21 dimensions (**Table 1**) indicated that teachers, on average, were classified in the “no symptoms” category for depression, “mild” for anxiety, and “no symptoms” for stress. However, analysis of individual category distribution (**Table 2**) showed that 18.5% of schoolteachers presented symptoms of possible depression, 59.3% anxiety, and 40.7% stress.

**Table 1:** Sociodemographic characteristics, mental health, and salivary cortisol of participants.

Variables	Mean	SD	Inferior CI95%	Inferior CI95%
Age (years)	39.63	14.38	33.94	45.32
Depression	3.22	3.08	2.00	4.44
Anxiety	4.63	3.44	3.27	5.99
Stress	6.89	4.33	5.18	8.60
Cortisol PM nmol/L	36.2	25.1	26.2	46.1

SD, standard deviation; PM, post meridiem; nmol/L, millimoles per liter.

**Table 2:** Frequency and percentages of schoolteachers presenting symptoms of/possible depression, anxiety, and stress.

Dimension	Categories	n	%
Depression	None	22	81.5
	Mild	1	3.7
	Moderate	3	11.1
	Severe	1	3.7
	Extremely severe	0	0
Anxiety	None	11	40.7
	Mild	5	18.5
	Moderate	6	22.2
	Severe	1	3.7
	Extremely severe	4	14.8
Stress	None	16	59.3
	Mild	4	14.8
	Moderate	5	18.5
	Severe	1	3.7
	Extremely severe	1	3.7

n, frequency; %, percentage.

**Figure 1:**
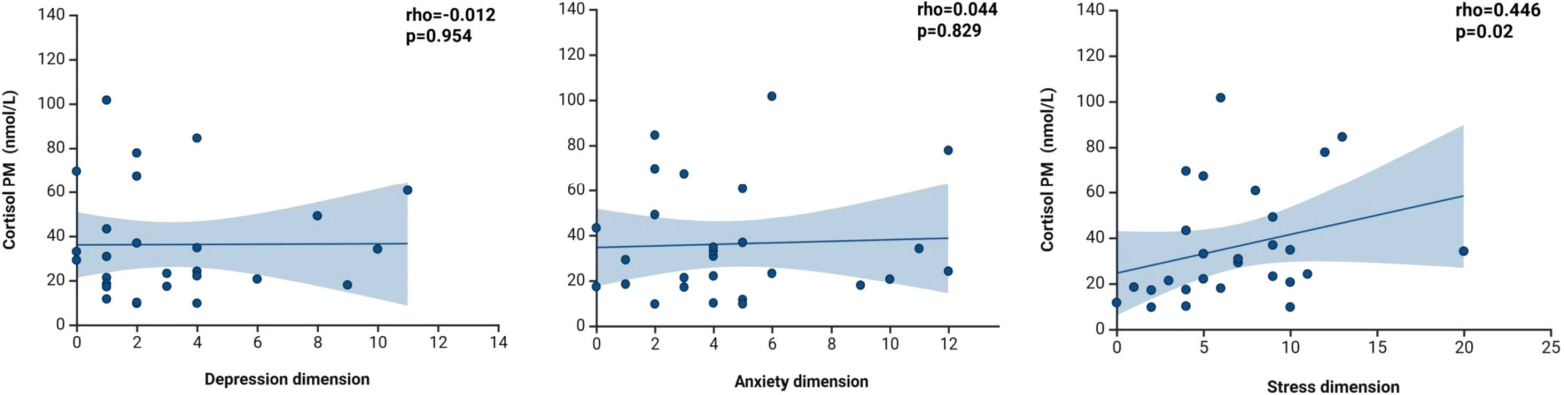
Correlation between post meridiem salivary cortisol and depression, anxiety, and stress dimensions.

Regarding PM salivary cortisol, the mean level was 36.2 nmol/L, and no participant presented values within the normal range (<6 nmol/L). Spearman correlation between cortisol levels and DASS-21 dimensions is shown in **Figure 1**. Results revealed a significant positive correlation between PM salivary cortisol and perceived stress (Rho = 0.446, p < 0.05). In addition, an extreme value was observed in the stress dimension (DASS-21 stress score = 20). For the extreme value, a correlation analysis was performed without it, and the correlation remained statistically significant removal of the extreme value (ρ = 0.449, p = 0.021). No significant correlations were observed between cortisol levels and the dimensions of depression or anxiety.

## Discussion

Research on teacher stress and its relationship with biological markers such as cortisol is essential for understanding the underlying mechanisms of mental health problems affecting education professionals. The positive correlation observed between PM salivary cortisol levels and perceived stress suggests a direct connection between subjective stress perception and the activation of the hypothalamic–pituitary–adrenal (HPA) axis, the primary stress response system (37). On the other hand, the absence of significant correlations between cortisol and the depression and anxiety dimensions indicates that perceived stress, measured through the DASS-21, captures a specific facet of teachers’ emotional experience, more closely related to the physiological stress response (38). This reinforces the utility of the DASS-21 as a monitoring tool for stress in this population (39, 40) although we acknowledge it is not a clinical instrument for diagnoses.

It is noteworthy that all teachers in the study presented PM cortisol levels above the established cutoff and although there is currently no consensus on the clinical relevance of this salivary analyte, the results do highlight that occupational stressors in the educational setting manifest in abnormal stress physiology and suggest that strategies to mitigate this adverse impact should be considered (41). The unusually high cortisol levels shown by the participants in the present study could represent a regional characteristic of Chilean schoolteachers, but we cannot confirm this without a description of baseline salivary cortisol levels at bedtime in the general Chilean population. Although the sample is small, these results align with previous research reporting a high prevalence of stress among teachers (4, 42, 43). The nature of teaching, characterized by high demands and multiple roles, explains why this population experiences elevated occupational stress levels (44). Recent studies also indicate that a considerable proportion of teachers report chronic stress, associated with physical and mental health problems (3, 45). In Chile, teachers’ mental health has systematically deteriorated in recent years (4, 46).

The DASS-21 scale is a reliable tool for assessing mental health in diverse contexts (4, 47). While Its validity and reliability in non-clinical samples and among teachers have been widely demonstrated, it is not a diagnostic clinical instrument (48, 49). Although average scores indicated mild or absent symptoms, individual analysis revealed a significant prevalence of depression, anxiety, and stress, highlighting the need for a detailed analysis of clinical categories. Early recognition of stress and the development of coping skills are essential to promote teachers’ health and well-being, positively impacting their performance and educational quality (50). Conversely, chronic exposure to stress and elevated cortisol levels has been associated with cardiovascular deterioration and neurodegeneration (51, 52).

Our results suggest that the stress dimension of the DASS-21 is a promising instrument for detecting and monitoring teacher stress, facilitating the implementation of timely intervention strategies. Study limitations include its cross-sectional design, which prevents establishing definitive causal relationships, and the non-probabilistic sampling, which could limit the generalizability of the findings. Additionally, the relatively small effective sample size for correlation analyses (n = 27) may have limited statistical power, increasing the risk of type II errors for small-to-moderate correlations. Therefore, the observed association between bedtime salivatory cortisol and perceived stress (Spearman’s ρ = 0.45; p < 0.05) should be interpreted as exploratory and warrants replication in larger samples. In this sense, as a pilot field study, a wide age range, and an uneven sex distribution, we were unable to formally assess the influence of age and sex on pre-bedtime salivary cortisol variability; therefore, residual confounding or effect modification by these factors cannot be ruled out and should be addressed in the future, adequately powered and sex-balanced studies. Moreover, the collection of cortisol at a single time point (before bedtime) does not capture the full circadian dynamics of the HPA axis. Furthermore, the exclusive use of the DASS-21 may not encompass all specific occupational stressors, such as workload or organizational climate (6, 7). Despite these limitations, the study contributes significantly to understanding the relationship between perceived stress and physiological biomarkers in the educational context.

## Conclusion

The present study demonstrates a significant association between perceived stress and PM salivary cortisol levels in teachers, indicating that the subjective perception of stress is reflected in a measurable physiological response. These findings highlight the need to address teacher stress through prevention, monitoring, and intervention strategies that consider both psychological and physiological aspects, in order to promote health and well-being within the educational setting.

## Data Availability

The raw data supporting the conclusions of this article will be made available by the authors, without undue reservation.
